# Liver histology in asymptomatic Hepatitis-C virus positive blood donors

**DOI:** 10.12669/pjms.341.14361

**Published:** 2018

**Authors:** Muhammad Anwar, Qamar Jamal, Waqaruddin Ahmed

**Affiliations:** 1 Dr. Muhammad Anwar, M.Phil (Pathology), Department of Pathology, BMSI, Jinnah Post Graduate Medical Centre, Karachi, Pakistan; 2 Dr. Qamar Jamal, PhD. (Pathology), Department of Pathology, Ziauddin Medical University, Clifton, Karachi, Pakistan; 3 Dr. Waqaruddin Ahmed, MD, Department of Gastroenterology, Pakistan Health Research Council, Jinnah Post Graduate Medical Centre, Karachi, Pakistan

**Keywords:** Family Blood Donors, Chronic HCV Infection, Normal ALT, Liver Histological changes

## Abstract

**Objective:**

To assess the Histological alteration of liver in asymptomatic HCV PCR positive family blood donors.

**Methods:**

This is a prospective cross sectional study carried out at Blood Transfusion Services, Clinical & Molecular Laboratory and Pakistan Health Research Council, Jinnah Postgraduate Medical Centre, Karachi from January 2011 to December 2016. One hundred thirteen (113) Anti HCV and HCV RNA positive family blood donors with normal or slightly raised ALT during three consecutive bimonthly visits were included. After taking consent liver biopsy was done to see the histopathological changes in these study participants. The software program SPSS for windows (Ver.19) was utilized for all statistical analysis.

**Results:**

One hundred thirteen blood donors who were Anti HCV and HCV RNA positive were included. Ninety eight were male and 15 Female with a mean age of 32.3±6.94 years. Nineteen (16.8%) had significant inflammation of grade 2-3. Nine (8.0%) had significant fibrosis and steatosis was seen in 65 (57.5%) cases. Cirrhosis or advanced liver disease was not found in this series.

**Conclusion:**

HCV infected individual with normal ALT level having good physical health, without any sign of advanced liver disease on ultrasonography and routine laboratory investigation like AST, Gamma GT, Alkaline Phosphatase, serum albumin, A/G ratio, platelet count and PT, INR might be highly helpful to predict mild or progressive liver disease. Ultimately it reduces the need of liver biopsy, an invasive procedure having significant complications.

## INTRODUCTION

Chronic HCV infection is a major health problem. Globally about 71 million people have this infection and around 399,000 people die every year due to chronic HCV infection related liver complication. The prevalence of chronic HCV infection in South East Asia is 0.5% in general population and approximately 10 million people have this infection^1^ while Pakistan have world second highest prevalence, 2^nd^ to Egypt, overall 6.7% population having HCV infection in this country.^2^

Around 20% to 30% Patient with chronic HCV infection have normal ALT level.^3^ It is a generally considered that Patients with normal ALT have lesser degree of inflammation & fibrosis and slower progression of disease as compared to cases with higher liver enzymes.^4^ Majority of the HCV carrier with normal ALT have normal to mild inflammation & fibrosis on liver biopsy while some studies have shown contradictory observations like so called “Healthy HCV Carrier” may have advanced fibrosis or even cirrhosis and Hepatocellular Carcinoma.^5^

Normal ALT level ranges between 7-56 U/L with slight variation in individual laboratory's reference values and ALT level >56 U/L considered high and abnormal.^6^-^7^ Persistently normal ALT (PnALT) defined as ALT level less than upper normal limits on three bimonthly consecutive serum levels for the period of six month.^8^ Indeed the accuracy of normal ALT to predict normal histology or any change related to HCV infection is still vague.^9^ Considering the truly healthy HCV positive blood donors, this study was conducted in healthy family blood donors who are incidentally found anti HCV reactive on Screening and also having HCV RNA positive, with normal ALT. Liver biopsy always have some risk and complication as 84% have mild discomfort, 20% have pain, 0.3% have serious complication while 0.01% reported death.^10^

With the realization about limitation of liver biopsy hepatologists have increased their efforts to develop non-invasive tools for assessment of liver inflammation and fibrosis.^11^,^12^ Many research studies have proven that chronic HCV infection associated liver fibrosis is continuous dynamic as well as reversible process, needs frequent assessment for fibrosis stage before and during antifibrotic therapy. Considering these fact efforts have been made to develop simple serum markers individually as well as in panels to achieve this task that will definitely reduce the demand of liver biopsy that is invasive procedure with significant complication.^13^,^14^

## METHODS

In this prospective cross sectional study, family blood donors, HCV antibody reactive on routine transfusion transmitted infections (TTI) screening. HCV RNA PCR was done of these Anti HCV reactive cases and those who found positive, finally included in this study. These study participants followed for six months to find out the ALT levels during three consecutive bimonthly tests. All research work done at Jinnah Postgraduate Medical Centre, Karachi during the period of January 2011 to December 2016. Transfusion transmitted infections screening include HCV Ab, HBs Ag, HIV Ab, Syphilis and ICT Malaria done at the department of Blood transfusion services. Complete Blood Count, Liver Function Tests, Prothrombin time, Activated Partial Thromboplastin Time, International Normalize Ratio, Albumin Globulin Ratio, Aspartate Aminotransferase, Alanine Aminotransferase, Gama-Glutamyl transferase, Random Blood Sugar and HCV RNA Polymerase Chain Reaction (Viral Load) performed at Clinical & Molecular Laboratory while Ultra Sonography at department of Radiology. Liver Biopsy carried out at Pakistan Health Research Council. Ethical review committee of Jinnah Postgraduate Medical Centre approved this study. Patients co-infected with HBV or HIV and with Diabetes, Hypertension, Joint pain and other chronic illness, and those with recent or past history of alcohol intake, and I/V drug use were excluded from the study. Written informed consent was received from each case.

### Liver Biopsy

A Percutaneous liver biopsy was performed with 16-18 gauge modified Menghini aspiration needle (SURECUT TSK Japan) with core of at least 10-15 mm in length or minimum five portal areas were considered suitable for interpretation. Liver biopsies having significant pathology with even less number of portal tracts were also included in the study.

Tissue was fixed in formalin, paraffin embedded and processed for light microscopy. Slides were stained with Haematoxylin and eosin stain along with Masson Trichrome helpful in evaluating pattern of fibrosis. Pathologist was unaware of clinical as well as biochemical data. Histological scoring of grading and fibrosis done on metavir scoring system.

Each specimen was graded on a four point scale depending upon the severity of focal lobular necro inflammation and piece meal necrosis. A0= No histological activity, A1= Mild activity, A2= Moderate activity, and A3= Severe Activity. Fibrosis was staged on five point scale. F0= No fibrosis, F1= Portal fibrosis without septa, F2= Portal fibrosis with rare Septa, F3= Numerous Septa without cirrhosis, F4= Cirrhosis

There is an intra and inter observer variability regarding activity grade & fibrosis stage on liver biopsy, however fibrosis beyond portal tract is considered F3 when 50% or more portal tract showed Fibrosis Septae beyond portal tracts. On the basis of necro-inflammatory grade and fibrosis stage these patients further divided into two severity groups. Minimal disease <F2 &<A2 while significant disease ≥F2 and ≥A2.

### Statistical Analysis

The data analysis was done using computer package SPSS (Statistical Package for Social Science) version 19.0. Clinical characteristics were summarized in terms of frequencies and percentages for qualitative/categorical variables i.e. gender, severity of inflammation, fibrosis, presence of steatosis, portal inflammation, lobular necrosis, mean ± S.D for quantitative/continuous variables i.e. age, weight, biochemical parameters (bilirubin, ALT, AST, Alkaline phosphates, Gamma GT, total protein, albumin, globulin, AG ratio, prothrombin time, Internal normalized ratio and platelet count. Statistical comparison performed using student t-test (independent test) for

**Fig. 1:**
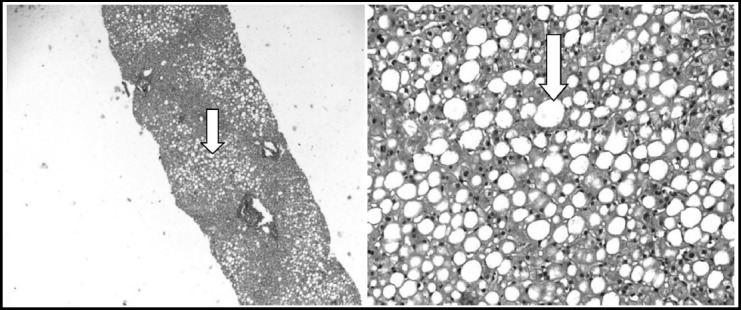
Low & High power view of grade 2-3 steatosis (Haemotoxylin & Eosin Stain x 100/400).

Quantitative/continuous variables and Chi-square test for qualitative/categorical variables between groups. In all statistical analysis only p-value <0.05 was considered significant.

## RESULTS

A total of 113 subjects (98 male and 15 female) were included in the study, with a mean age of 32.3 ± 6.94 years. Nineteen (16.8%) had significant inflammation (Grade 2-3), 9 (8.0%) had significant fibrosis (Stage 2-3) and Steatosis was seen in 65 (57.5%) cases.

### Hepatic inflammation

A significant relationship was seen with severity of fibrosis, presence of steatosis, mean portal tract level, portal inflammation, lobular necrosis and piecemeal necrosis but there was no significant relationship between the severity of inflammation, age, gender and biochemical parameters (Table-I).

**Table-I:** Patients Characteristics with Inflammation of liver.

Parameters	Significant Inflammation (Grade 2-3) (n=19)	Insignificant Inflammation (Grade 0-1) (n=94)	P-value
Male	16 (84.2%)	82 (87.2%)	0.723
Female	3 (15.8%)	12 (12.8%)	
Age (years)	31.9 ± 7.27	32.4 ± 6.91	0.790
*Biochemical parameters*			
Bilirubin – Total	1.15 ± 0.46	1.06 ± 0.54	0.455
Bilirubin – Direct	0.63 ± 0.31	0.50 ± 0.28	0.111
ALT (IU/L)	46.4 ± 11.75	44.6 ± 13.31	0.553
AST (IU/L)	39.6 ± 14.13	38.5 ± 12.56	0.760
Alk. Phos	202 ± 51.0	208 ± 63.7	0.685
Gama GT	23.9 ± 9.71	25.8 ± 15.8	0.495
Total protein	8.16 ± 0.85	8.43 ± 0.76	0.215
Albumin	4.66 ± 0.44	4.59 ± 0.38	0.481
Globulin	3.49 ± 0.98	3.84 ± 0.73	0.157
AG Ratio	1.50 ± 0.70	1.25 ± 0.33	0.142
Prothrombin time	12.7 ± 1.53	12.0 ± 1.72	0.647
Internal Normalized Ratio	1.06 ± 0.08	1.04 ± 0.17	0.547
Platelet count (ul)	199 ± 77.5	211 ± 66.5	0.578
*Severity of Fibrosis*			
Significant (Fibrosis 2-3)	9 (47.4%)	0	0.001
Insignificant (Fibrosis 0-1)	10 (52.6%)	94 (100.0%)
*Presence of Steatosis*			
Present	16 (84.2%)	49 (52.1%)	0.010
Absent	3 (15.8%)	45 (47.8%)
Portal Tracts	11.1 ± 4.11	8.97 ± 4.34	0.050
*Portal Inflammation*			
Significant (2-3)	17 (89.5%)	2 (2.1%)	0.001
Insignificant (1)	2 (10.5%)	92 (97.9%)
*Lobular Necrosis*			
Significant (1-2)	18 (94.7%)	52 (55.3%)	0.001
Insignificant (0)	1 (5.3%)	42 (44.7%)
*Piecemeal Necrosis*			
Significant (1-2)	17 (89.5%)	70 (74.5%)	0.001
Insignificant (0)	2 (10.5%)	24 (25.5%)	

**Fig. 2:**
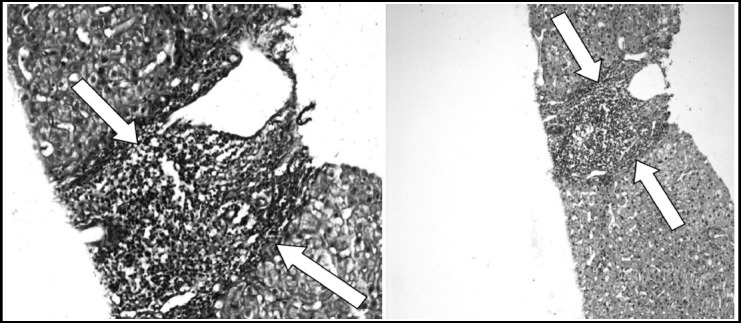
Portal to central septae, Masson Trichrome, Haemotoxylin & Eosin Stain x 200.

### Hepatic fibrosis

A significant relationship was seen with severity of inflammation, portal inflammation, lobular necrosis, and piecemeal necrosis but there was no significant relationship between the severity of fibrosis, age, gender and biochemical parameters (Table-II).

**Table-II:** Patients Characteristics with Liver Fibrosis.

Parameters	Significant Fibrosis (Stage 2-3) (n=9)	Insignificant Fibrosis (Stage 0-1) (n=104)	P-value
Male	7 (77.8%)	91 (87.5%)	0.410
Female	2 (22.2%)	13 (12.5%)	
Age (years)	31.3 ± 8.12	32.4 ± 6.86	0.705
*Biochemical parameters*			
Bilirubin – Total	1.26 ± 0.60	1.06 ± 0.52	0.378
Bilirubin – Direct	0.61 ± 0.38	0.51 ± 0.28	0.495
ALT (IU/L)	50 ± 7.8	44 ± 13.3	0.097
AST (IU/L)	40 ± 17.4	38 ± 12.4	0.785
Alk. Phos	206 ± 45.4	207 ± 62.9	0.934
Gama GT	23.0 ± 9.05	25.7 ± 15.4	0.440
Total protein	7.9 ± 0.76	8.4 ± 0.77	0.117
Albumin	4.6 ± 0.46	4.6 ± 0.39	0.844
Globulin	3.3 ± 0.58	3.8 ± 0.77	0.144
AG Ratio	1.5 ± 0.61	1.3 ± 0.40	0.260
Prothrombin time	12 ± 1.6	12 ± 1.7	0.919
Internal Normalized Ratio	1.02 ± 0.07	1.05 ± 0.16	0.253
Platelet count (ul)	182 ± 46.6	211 ± 69.5	0.107
*Severity of Inflammation*			
Significant (Grade 2-3)	9 (100%)	10 (9.6%)	0.001
Insignificant (Grade 0-1)	-	94 (90.4%)
*Presence of Steatosis*			
Present	7 (77.8%)	58 (55.8%)	0.200
Absent	2 (22.2%)	246 (44.2%)	
Portal Tracts	9.7 ± 3.84	9.3 ± 4.41	0.791
*Portal Inflammation*			
Significant (2-3)	8 (88.9%)	11 (10.6%)	0.001
Insignificant (1)	1 (11.1%)	93 (89.4%)
*Lobular Necrosis*			
Significant (1-2)	9 (100%)	61 (58.7%)	0.001
Insignificant (0)	-	43 (41.3%)
*Piecemeal Necrosis*			
Significant (1-2)	9 (100%)	78 (75.0%)	0.001
Insignificant (0)	-	26 (25.0%)	

### Hepatic Steatosis (Fat accumulation)

A significant relationship was found with severity of inflammation but there was no significant relationship between the presence of steatosis, age, gender and biochemical parameters (Table-III).

**Table-III:** Patients Characteristics with Steatosis in Liver.

Parameters	Steatosis Present (n=65)	Steatosis Absent (n=48)	P-value
Male	53 (81.5%)	45 (93.8%)	0.059
Female	12 (18.5%)	3 (6.3%)	
Age (years)	32.7 ± 6.94	31.8 ± 6.97	0.528
*Biochemical parameters*			
Bilirubin – Total	1.05 ± 0.57	1.12 ± 0.45	0.448
Bilirubin – Direct	0.49 ± 0.28	0.56 ± 0.29	0.217
ALT (IU/L)	47 ± 12.4	41 ± 13.5	0.091
AST (IU/L)	40 ± 12.7	37 ± 12.7	0.214
Alk. Phos	210 ± 68.2	202 ± 51.6	0.467
Gama GT	24.8 ± 12.7	26.4 ± 17.6	0.613
Total protein	8.3 ± 0.72	8.4 ± 0.86	0.843
Albumin	4.6 ± 0.41	4.5 ± 0.37	0.195
Globulin	3.7 ± 0.77	3.8 ± 0.81	0.403
AG Ratio	1.33 ± 0.46	1.25 ± 0.37	0.300
Prothrombin time	12.4 ± 1.59	11.8 ± 1.01	0.078
Internal Normalized Ratio	1.06 ± 0.19	1.04 ± 0.12	0.434
Platelet count (ul)	204 ± 67.2	215 ± 69.6	0.373
*Severity of Inflammation*			
Significant (Grade 2-3)	16 (24.6%)	3 (6.2%)	0.010
Insignificant (Grade 0-1)	49 (75.4%)	45 (93.8%)
*Fibrosis*			
Significant (Stage 2-3)	7 (10.8%)	2 (4.2%)	0.200
Insignificant (Stage 0-1)	58 (89.2%)	46 (95.8%)	
Portal Tracts	9.3 ± 4.68	9.4 ± 3.93	0.884
*Portal Inflammation*			
Significant (2-3)	14 (21.5%)	5 (10.4%)	0.254
Insignificant (1)	51 (78.5%)	43 (89.6%)	
*Lobular Necrosis*			
Significant (1-2)	43 (66.2%)	27 (56.3%)	0.423
Insignificant (0)	22 (33.8%)	21 (43.8%)
*Piecemeal Necrosis*			
Significant (1-2)	49 (75.4%)	38 (79.2%)	0.718
Insignificant (0)	16 (24.6%)	10 (20.8%)	

## DISCUSSION

This study was conducted in family blood donors who have normal or slightly raised ALT level with anti-HCV reactive on routine TTI screening. Majority of the cases (87%) were male between the ages of 20 to 55 years, with a mean age ± S.D 32.3 ± 6.94 years, which is the normal trend of blood donors in our country. In this study group 19 (16.8%) had significant inflammation (Grade 2-3), 9 (8%) had significant fibrosis (Stage 2-3) and steatosis was seen in 65 (57.5%). None of the case had cirrhosis or advanced liver disease. Similar kind of study done in Voluntary Blood Donors by de Santana et al. revealed that 18% had minimum histological alteration, 49% had chronic hepatitis with minimum to moderate activity while cirrhosis was detected in 14% individual^15^, while Jamal et al. has described similar results as our study.^16^ Most of the studies are contradictory to our results, like Khattab H et al. found that 35% of the infected patients studied have advanced liver disease and about 6% have cirrhosis.^17^ Akbar HO et al. found 8 (15.4%) patients had normal liver biopsy and 44 (86.6%) had abnormal liver histology. Out of 44 patients, 23 (44.2%) had mild histology (F_1_-F_2_), 15 (28.8%) patients had severe histological changes (F_3_), 2 (3.8%) patients had cirrhosis (F_4_), 4 (7.7%) had non-specific changes (A_1_/F_0_).^18^ Memon MS et al. obtained 55 liver biopsies and found around 60% patients had inflammation equal to or greater than grade 2 while 43.6% patients showed fibrosis equal to or greater than stage (Metavir F≥2), cirrhosis was seen in 7.3% of patients.^19^ Roshan B et al. reported that fibrosis and necro inflammation were comparable among PnALT and abnormal ALT groups. He also found that advanced fibrosis was more common in PnALT group as compared with Elevated ALT group.^20^

In our study we found 94 cases (83.2%) had insignificant inflammation (grade 0-1) and 104 (92.0%) insignificant fibrosis (stage 0-1). We found lesser degree of inflammation and fibrosis contradictory to the other studies as most of the studies done on chronic HCV patients instead of blood donors, without considering general physical health, co- infections or chronic illness, environmental factors like alcohol or I/V drug use that can aggravate disease progressions. Moreover we excluded all participants initially having features of advanced liver disease on laboratory investigations and ultrasonography even they have persistently normal ALT level.

## CONCLUSION

Our study results reveal that patients having normal ALT level, no clinical sign of advanced liver disease on liver specific lab investigations and ultrasonography can be highly helpful to predict mild liver disease. Pakistan is among the highest prevalence of chronic HCV infection in the world and also comes in low human development index category. Taking into consideration the high disease burden and non-affordability of general population we recommend that simple routine liver specific lab investigations, ultrasonography, and overall health assessment can significantly minimize the requirement of liver biopsy. It is also helpful to prioritize the treatment options in resources constraint society.

**Fig. 3:**
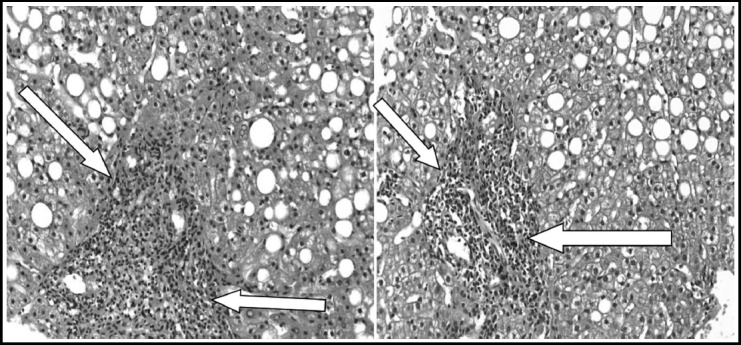
Portal expansion, moderate lymphoid aggregate with marked interface hepatitis. Haemotoxylin & Eosin Stain x 400.

### Authors Contribution

**MA:** Conceived designed, statistical analysis and editing of manuscript.

**QJ:** Critical analysis, review and final approval of manuscript.

**WA:** Data collection & liver biopsies.
